# Laparoscopic treatment of a solitary fibrous tumor originating in the cystic plate

**DOI:** 10.1186/s40792-018-0556-7

**Published:** 2018-12-29

**Authors:** Yu Kumagai, Shuichi Fujioka, Takeyuki Misawa, Hiroaki Kitamura, Masafumi Suzuki, Katsuhiko Yanaga

**Affiliations:** 1 Department of Surgery, The Jikei University Kashiwa Hospital, 163-1, Kashiwashita, Kashiwa City, Chiba 277-8657 Japan; 2 Department of Pathology, The Jikei University Kashiwa Hospital, Chiba, 277-8657 Japan; 3 Department of Surgery, The Jikei University School of Medicine, Tokyo, 105-8461 Japan

**Keywords:** Solitary fibrous tumor, Cystic plate, Laparoscopic resection, Gallbladder

## Abstract

**Background:**

Solitary fibrous tumor (SFT) is a rare mesenchymal tumor originating from the tissue underlying the mesothelial layer of the pleura or mediastinum. Other reported sites include the upper respiratory tract, orbit, thyroid, peritoneum, and central nervous system.

**Case presentation:**

We describe a case of a 7 cm SFT that originated in the cystic plate. A liver tumor was an incidental finding in a 49-year-old woman during a regular radiological checkup for uterine fibroids. Imaging revealed a well-circumscribed solid mass between the gallbladder and liver. Intraoperative laparoscopy identified a soft tumor that had progressively expanded behind the gallbladder which was easily separated from the Laennec’s capsule of the liver. Hematoxylin and Eosin and immunohistochemical staining of the tumor tissue found both tangled and patterned arrangements of spindle cells consistent with a SFT derived from the subserosal layer of the gallbladder.

**Conclusions:**

To the best of our knowledge, this is the first report of a SFT originating in the cystic plate.

## Introduction

Solitary fibrous tumor (SFT) is a rare mesenchymal tumor originating from the tissue underlying the mesothelial layer of the pleura or mediastinum [1]. Other reported sites include the upper respiratory tract, orbit, thyroid, peritoneum, and central nervous system [1, 2]. Most patients are asymptomatic because of slow tumor growth. Symptoms generally develop only after the lesion has become very large [2]. Clinical or radiological diagnosis of SFTs is difficult because specific symptoms and signs are lacking, and preoperative fine needle aspiration cytology is not always convincing. Laparotomy and pathological evaluation are usually performed for diagnosis. We describe a rare case of SFT originating in the cystic plate which was successfully removed laparoscopically.

## Case presentation

**Fig. 1:**
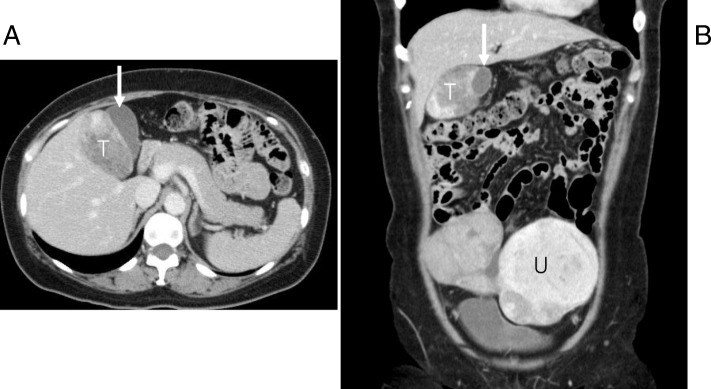
**a**, **b** Dynamic computed tomography demonstrated a hypodense tumor (T) measuring approximately 7.0 cm in diameter beside the gallbladder (white arrow) apart from the uterine fibrosis (U)

**Fig. 2:**
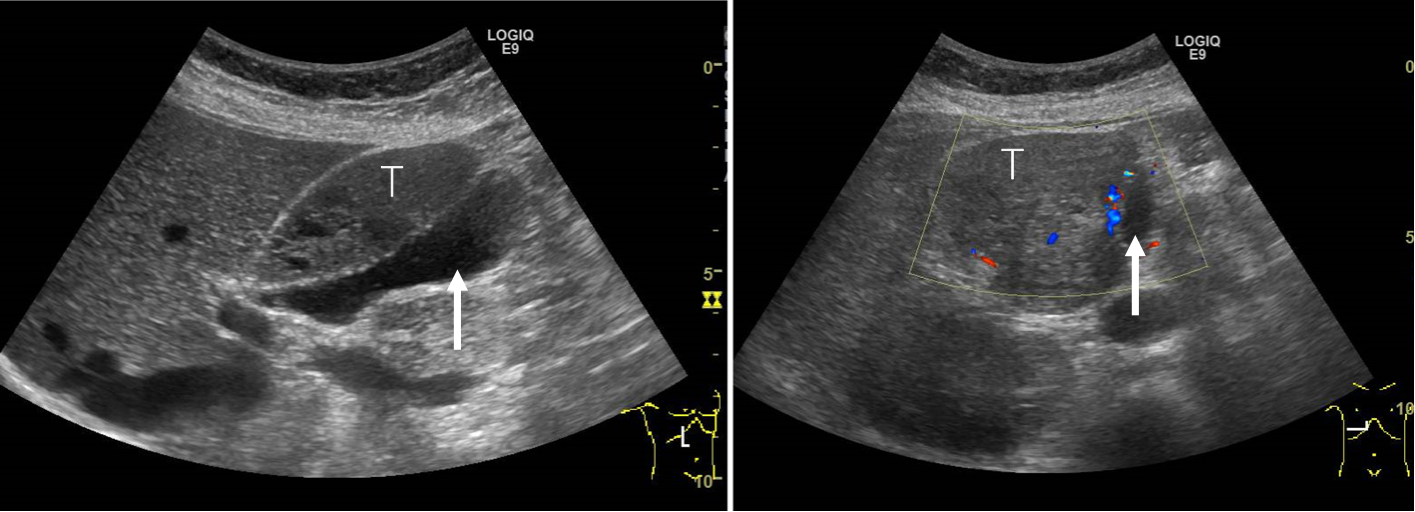
Abdominal ultrasonography showed a tumor (T) with a clear border between the gallbladder (white arrow) and the liver

**Fig. 3:**
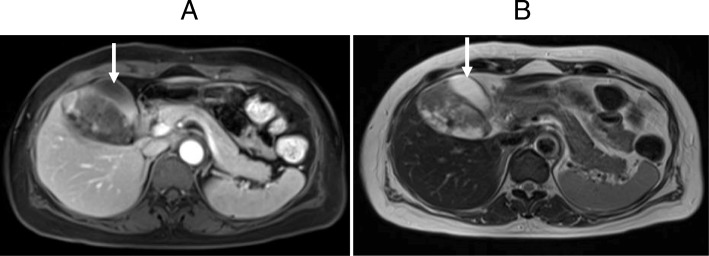
Abdominal magnetic resonance imaging found a tumor between the gallbladder (white arrow) and the liver. **a** T1 W1. **b** T2 W1

**Fig. 4:**
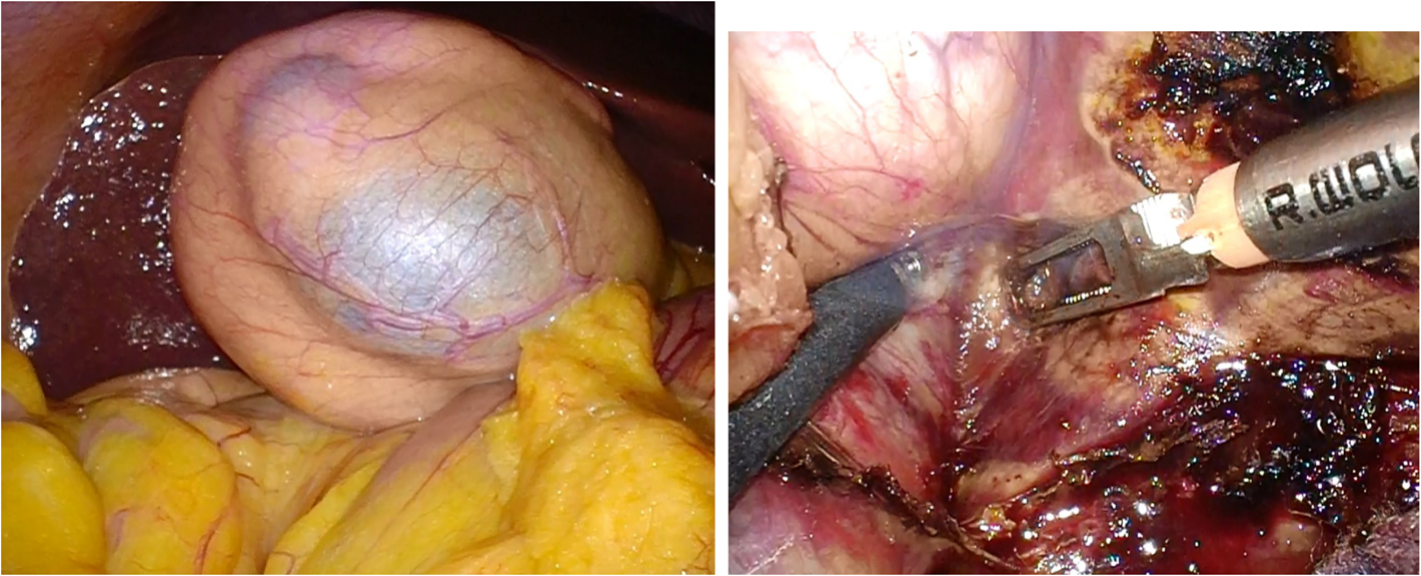
Intraoperative laparoscopy found a relatively soft tumor behind the gallbladder with dorsal compression of the liver

Abdominal computed tomography for regular monitoring of uterine fibrosis in a 49-year-old woman found a hypodense lesion 7 cm in diameter with a clear boundary near the gallbladder (Fig. 1a, b). No obvious change in size and internal density of uterine fibrosis had been observed during these 5 years (Fig. 1b). She had no previous history of alcohol or drug abuse. Ultrasound revealed a well-defined, non-calcified tumor between the gallbladder and liver (Fig. 2). Magnetic resonance imaging demonstrated a hypointense tumor that compressed the gallbladder and liver on precontrast T1 mapping (Fig. 3a). T2-weighted images revealed hyperintense tumor with delayed enhancement on arterial phase and portal venous phase (Fig. 3b) followed by a delayed washout on the hepatocyte phase. Clinical evaluation and laboratory results were nonspecific, and serum tumor markers including carcinoembryonic antigen, cancer antigen (CA) 19-9, alpha-fetoprotein (AFP) and squamous cell carcinoma antigen were within their normal ranges. The findings were consistent with a gastrointestinal stromal tumor (GIST) or another benign tumor originating from the liver or gallbladder. Accordingly, we planned firstly laparoscopic partial hepatectomy concomitant with gallbladder resection. Lymphadenectomy among the hepatoduodenal ligament were also planned under the situations where malignant tumor was suspected intraoperatively. Under the laparoscopic view, a soft tumor had expansively progressed behind the gallbladder, with dorsal compression of the liver (Fig. 4). The tumor was easily separated from Laennec’s capsule of the liver at the gallbladder neck and body without adhesion, which indicates the tumor is benign. Therefore, operative policy changed to tumor enucleation with cholecystectomy. Frozen sections including the surgical margin of the cystic duct were negative for tumor cells. As the tumor was moderately attached to the liver at the fundus of the gallbladder, the liver parenchyma was partially resected en bloc to ensure that the surgical margins were free of tumor tissue.

**Fig. 5:**
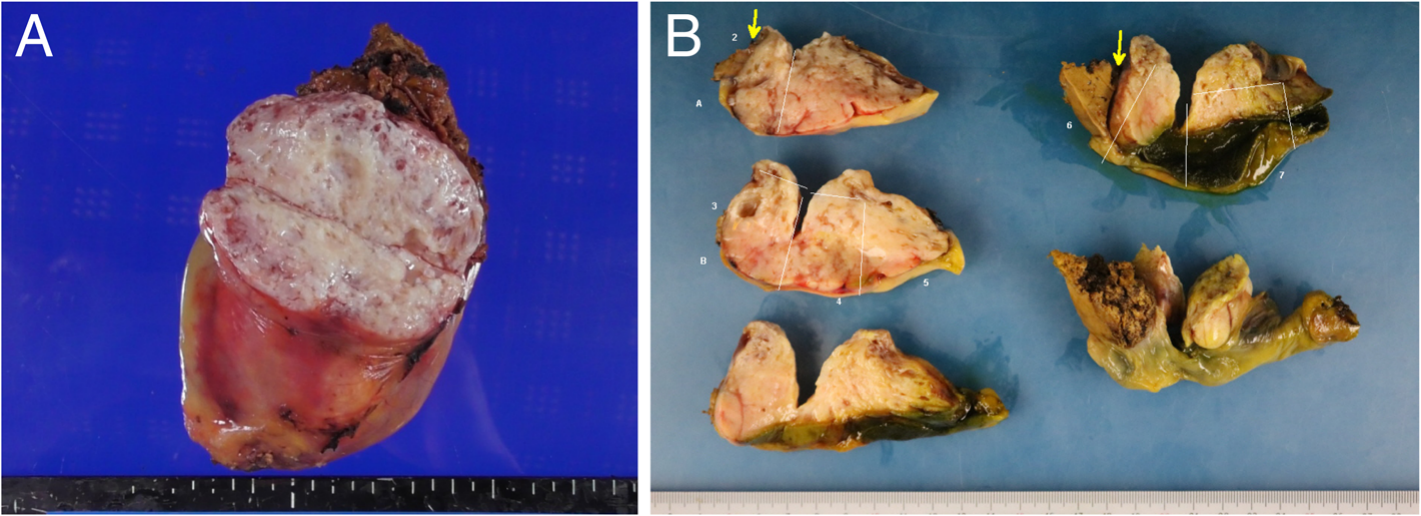
The resected specimen included a soft, clear-bordered white tumor between the liver tissue and gallbladder that originated from sub-serosal layer of the gallbladder (**a**, **b**)

**Fig. 6:**
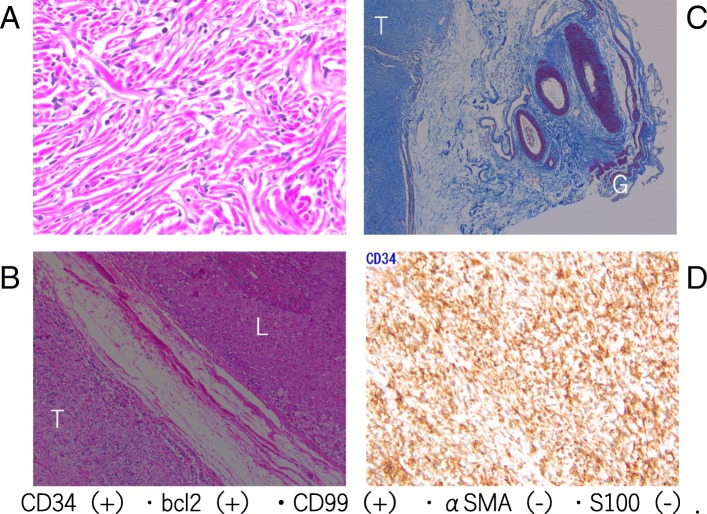
Histological findings of the resected specimen show **a** the microscopic appearance of Hematoxylin and Eosin-stained spindle-shaped tumor cells with elongated nuclei (40×) and **b** of Masson’s Trichrome staining (40×). **c** The tumor (T) was free from the gallbladder (G), and from the liver (L). **d** Immunohistochemical staining for CD34 (100×)

The resected specimen included soft and white tumor tissue with clear boundaries that was located between the liver and gallbladder (Fig. 5). Pathological examination following Hematoxylin and Eosin and immunohistochemical staining of tumor specimens was consistent with SFT. The specimens included spindle-shaped tumor cells with elongated nuclei (Fig. 6a), present in both tangled and patterned arrangements in the subserosal layer of the gallbladder (Fig. 6b, c). The tumor tissue had a storiform pattern with alternating hypocellular and hypercellular areas with some showing myxoid degeneration. The tumor cells were positive for CD34, CD99 and B-cell lymphoma (BCL)-2 and negative for S100 and alpha smooth muscle antigen (αSMA) staining (Fig. 6d), and few cells were positive for the cell proliferation marker Ki-67/MIB-1. The tumor cells were negative for SMA, keratin, cytokeratin (AE1/AE3), CD117, epithelial membrane antigen, and desmin. The pathological diagnosis was SFT originating from the cystic plate.

The postoperative course was uneventful. She was discharged on postoperative day 9. Chemotherapy was not considered necessary, and postoperative follow up at 18 months found the patient well without any sign of recurrence. Periodical check-up for uterine fibrosis by gynecologist is also continued.

## Discussion

SFT was first described in 1931 [3], which is a rare neoplasm primarily occurring in the pleura, but has been reported to originate in other organs [1, 2]. Liver involvement is uncommon [4], and a SFT originating from in the cystic plate has not been previously reported. Non-specific laboratory values and imaging characteristics make it difficult to distinguish SFTs that develop in the liver parenchyma from other tumors such as hepatocellular carcinoma, leiomyoma, sarcoma, sclerosed hemangioma, and inflammatory pseudotumor [5]. Histopathological evaluation is generally needed to confirm the diagnostic of SFT. CD34-positive fibroblast-like mesenchymal cells or dendritic interstitial cells are useful for diagnosis.

The treatment of SFTs is surgical resection, chemotherapy, and radiation therapy have not been shown to be effective [6]. In the current case, the tumor was diagnosed pathologically to have originated from the cystic plate, which is in continuity with the subserosal layer of the gallbladder. Previous cases of SFT of the liver have been reported to originate from the mesothelial tissues such as Glisson’s sheath or connective tissue, but proliferate as an intrahepatic mass that includes connective tissue, which is why SFTs of the liver have previously been detected as intraparenchymal masses [7]. To our knowledge, the current case is the first report of a SFT that derived from the cystic plate.

The prognosis of SFT is generally favorable, but some cases of malignancy have been reported, with a 10.8% recurrence [8]. As recurrent SFTs progress slowly, observation and possible re-operation can be chosen. Cellular atypia, high cellularity and mitotic rate, tumor necrosis, and positive tumor margins are reported to correlate with local recurrence. Complete resection of a tumor is believed to be the best prognostic indicator [9], but close follow-up is recommended even after successful resection because of possible recurrence. In the present case, while the tumor was intraoperatively thought to be a benign tumor such as a desmoid tumor, minimal surgical resection was chosen. Two-stage additional hepatectomy with lymphadenectomy of the hepatoduodenal ligament for the potentially residual tumor cells were considered, taking the permanent pathological diagnosis as SFT. Finally, careful follow-up has been chosen, because of the lack of evidence of desirable surgical margin as well as definite adjuvant therapy. Fortunately, she is still doing well until now, 17 months after surgery without any recurrences.

## Conclusions

To our knowledge, this is the first report of a SFT originating from the cystic plate. SFT should be included as a rare cause of liver tumors, which long-term follow-up is warranted.

## Abbreviations

SFT: Solitary fibrous tumor
